# Partial Tenotomy—A Radical Cure for Heterophoralgia

**Published:** 1893-07

**Authors:** C. H. Peete

**Affiliations:** No. 572 Mulberry Street; Macon, Ga.


### PARTIAL TENOTONY—A RADICAL CURE FOR HETEROPHORALGIA.

BY C. H. PEETE, M. D., MACON, GA.

This term, Heterophoralgia, being comparatively a new one, needs some explanation. To those who have not made themselves acquainted with the use of it, I will say that it is a name given by Dr. G. H. Price, of Nashville, Tenn., to a chain of symptoms caused by the expenditure of much nerve force, in order to maintain perfect binocular single vision, where there is a tendency of the visual lines to deviate from parallelism. These symptoms, such as dull heavy headaches, especially those through the temples and forehead, a blurring of the letters after reading a short while, and what is commonly known as sick headaches, are all representatives of heterophoralgia, and yet are frequently caused by an error of refraction, which error should be properly corrected by glasses. After correcting the existing errors of refraction, we should test the muscles to ascertain if the opposing ones are of equal strength. If not, we measure the amount of difference in degrees. Should that deviation be only of such an amount that can be corrected by prisms, we do so, if it cannot be corrected by the use of prisms or decentering the lens, we resort to our radical means of Partial Tenotomy.

This tendency to deviation may be in any direction. We have had names given it for the deviation in different directions, which express them exactly and nicely.

In order to discuss the subject intelligently, I must go a little farther in the examination of the cause which I claim calls for the operation. We know that where there is an actual turning of one eye in, out, up or down, that the deviation of the visual line from parallelism is so great that the nerve force allotted the muscles attached to the ball is not sufficient to maintain binocular single vision, so that not being able to maintain the single vision, the impression made on one eye is not noticed, consequently the unbalanced eye, finding itself unable to maintain its position, naturally turns or yields to the stronger of the four recti muslces, which carries it from parallelism of the visual lines in proportion to the superiority in strength over its opponent.

So it is where we have the condition called heterophoria only, that the deviation is of such an amount that with a considerable strain we can maintain single vision.

As it is with other muscles of the body, so it is with the recti muscles of the eye. Suppose two men of unequal strength are pulling on a beam which is fastened at its center, we can readily see that the one of inferior strength will sooner become exhausted, and it is then with considerable effort that he can maintain his position. We can also see that at the end of a given time the weaker man will be compelled to rest or he will fail to do his work. Now then, we know that if this is the case with men of unequal strength, it will also apply to muscles of unequal strength.

When this heterophoric condition exists, we know that there is a deviation of visual lines from parallelism, and that in order to *bring them to parallelism* there must be expended an additional amount of nerve force by the weaker muscle. This condition being constant, and unless relief comes to the overtaxed muscle, there will be as a result a train of symptoms named by Dr. Price as Heterophoralgia. Knowing the laws of refraction, we know that a condition of this kind can, to a certain degree, be corrected by the use of proper lenses; beyond that degree, I claim that the only radical cure is Partial Tenotomy.

The two muscles being of unequal strength it is our business to equalize them. There are different theories as to the cause of this deviation, one being that one of the muscles is over-developed, another being that one of the muscles is under or not sufficiently developed; the third theory is that the maculae are congenitally misplaced. As far as the treatment is concerned, they all amount to one and the same thing. The visual lines must be made parallel. In performing this little operation we give relief to many patients, who have been sufferers for years.

While the operation is being done by a great many, there are yet some of our leading ophthalmologists who do not practice it. It may be said that some object to it, merely from a point of prejudice, not having tried the operation or seen its results, while others are conscientious in their views and belief that relief can be gotten by the use of prisms or de-centering lenses.

In converging for the new point, the internal recti are brought into play, and they being the weaker muscles, it is with some effort they are able to maintain the position required for binocular single vision.

As this reserve force is brought out and used up to assist the internal recti in converging, so in proportion the disagreeable symptoms are increased. Heretofore, those who have suffered from this trouble and were not relieved by properly adjusted glasses, have been compelled to abandon in a great measure, their near work, in order to free themselves of this terrible suffering. The fact is that it is only within the last few years that we have been able to find the cause of them. Since the introduction of the Maddox Rod and double prism tests, we have been able to diagnose these deviations beyond a doubt.

Dr. Savage, in an article published in the *Ophthalmic Record*, of January, 1892, gives us a dividing line between operative and the non-operative cases, and to this article I refer you for that consideration. Whenever I find that by placing a plain red glass before one eye causes diplopia for distance, I operate at once, dividing just enough of the fibers of the stronger muscles to equalize the strength of it with that of its opponent, making the visual lines as near parallel as possible, then correct the remaining amount with glasses.

In my practice, I find that the majority of cases suffering from heterophoralgia are those of young adult life, or that they have been sufferers since that time. I account for that by the fact that we know in order to cause it, the patient must concentrate his vision on an object, and the length of time of this concentration governs the intensity of suffering.

In children the vision is never fixed on any point for a length of time, consequently these symptoms are not brought on. In young adults who have acquired the habit of hard study and constant close work, it occurs frequently, and you will find in most cases that it commenced with the time of their beginning the work or in a short time after. These muscle tests should be applied to every case who comes to us for refraction work, and when we find these deviations existing, we should resort to the radical relief, Partial Tenotomy.

The operation is too well known to need any comment.

No. 572 Mulberry Street.

